# Effectiveness of Music Interventions to Reduce Test Anxiety in Pharmacy Students

**DOI:** 10.3390/pharmacy9010010

**Published:** 2021-01-05

**Authors:** Suzanne Galal, Deepti Vyas, Rachelle Kisst Hackett, Ed Rogan, Chloe Nguyen

**Affiliations:** 1 Thomas J. Long School of Pharmacy, University of the Pacific, Stockton, CA 95211, USA; dvyas@pacific.edu (D.V.); erogan@pacific.edu (E.R.); h_nguyen83@u.pacific.edu (C.N.); 2 Benerd College, University of the Pacific, Stockton, CA 95211, USA; rhackett@pacific.edu

**Keywords:** music, test anxiety, stress, coping mechanisms, self-awareness

## Abstract

Background: The main objective of this pilot study was to evaluate the impact of a classroom activity involving music on anxiety associated with preparing for and taking an assessment. Methods: Two hundred and two (202) pharmacy students were randomly assigned to one of two conditions of the experimental study: active music playing (*n* = 103) versus passive music listening (*n* = 99). All students completed a pre-test, a mid-test, and a post-test including: an “Attitudes and Perceptions” survey, State-Trait Anxiety Inventory for Adults (STAI Survey), and a knowledge assessment. Data were analyzed to determine the impact each of the music interventions had on students’ test anxiety. Results: Both types of musical interventions produced similar results in terms of anxiety reduction. Faced with an upcoming test prior to the musical intervention, average state-trait anxiety scores increased; after the musical intervention, state-trait anxiety scores decreased. Conclusions: The use of music helped to reduce test anxiety, even after one brief musical intervention, regardless of whether students passively listened to music or actively played music.

## 1. Introduction

Test anxiety is multifactorial and can be defined as “the set of phenomenological, physiological, and behavioral responses that accompany concern about possible negative consequences or failure on an exam or similar evaluative situation” [1]. Test anxiety is a specific subset under the umbrella of symptoms characterized by the feelings of “anxiety”. Under the Diagnostic and Statistical Manual -IV (DSM-IV) test anxiety is most closely associated with the classification of social phobia, which is characterized by “a marked and persistent fear of social or performance situations in which embarrassment may occur.” [2]. In a study on components of test anxiety, Liebert and Morris pointed out that test anxiety encompasses two central aspects, cognitive factor (e.g., “worry” or “lack of confidence”) and emotionality (e.g., autonomic reactions that tend to occur under stress) [3]. Of the two aspects, cognitive factor is inversely related to performance expectancy: the more one is worried, the worse one performs [3]. As a result, test anxiety is associated with lower academic performance across all educational levels [4,5,6].

The curriculum for health care professionals is demanding and students are subjected to an overwhelming amount of stress secondary to a multitude of factors. These can include lack of sleep, diminished time for leisure, pressure to perform well on assessments, and the overabundance of knowledge to learn on any given day [7,8]. In a study conducted in 2013 at Alexandria University of Egypt, the prevalence of anxiety and depression within the medical and pharmacy student population was alarming [9]. Students were asked to complete the twenty-one-item Beck Anxiety Inventory (BAI) and the twenty-one-item Beck Depression Inventory (BDI) to measure anxiety and depressive symptoms of the students [10,11]. In the medical school, 43.9% (*n* = 72) of students suffered anxiety and 57.9% (*n* = 95) suffered depression. Within the pharmacy school, 29.3% (*n* = 48) of students reported anxiety and 51.1% (*n* = 84) reported depression [9]. In a systemic review and meta-analysis published in 2016, data were extracted from 167 cross-sectional studies (*n* = 116,628) and 16 longitudinal studies (*n* = 5728) from 43 countries showing a prevalence in depression or depressive symptom data in medical students of 27.2% [12]. The Beck Depression Inventory was used in 57 of the studies [12]. In another study led by Dawood et al., 277 nursing students were administered an anxiety inventory test; 65% (*n* = 181) experienced moderate to severe test anxiety and a statistically significant negative relationship was found between test anxiety score and students’ academic performance as measured by Grade Point Average (GPA) [13]. Sansgiry and colleagues examined factors related to test anxiety in pharmacy students and found that test competence and academic competence were the most significant predictors of test anxiety [14]. The authors emphasize the necessity of enhancing students’ competence not only with well-structured courses but with the incorporation of stress management strategies into the curriculum [14]. Reduction in stress is an important factor in allowing professional students to perform at their highest potential academically and professionally [15]. Nursing students reported lower test anxiety after completing a stress-management intervention program that included coping strategies related to time-management, testing skills, nutrition, exercise, relaxation methods, and cognitive control [16].

While not fully understood, previous research have found that music influences health via changes in neurochemical systems of a/dopamine and opioids, b/cortisol, corticotrophin-releasing hormone (CRH), andrenocorticotropic hormone (ACTH), c/serotonin and the peptide derivatives of proopiomelanocortin (POMC), and d/oxytocin [17]. In a systemic review of 44 studies assessing the biological impact of music listening in both clinical and nonclinical settings, 33 different biomarkers were considered in relation to music listening; of which, 13 biomarkers were significantly affected by music compared to a control condition [18]. The most analyzed biomarker was cortisol (35 out of 44 studies). In 14 of those studies, music led to either a higher decrease or lower increase in cortisol level than the control condition [18]. While music has long been used as a method to reduce stress in a wide range of settings and populations; the use of music in academia needs to be further explored [19,20,21,22,23]. Lilley and colleagues showed a correlation between music, test anxiety, and grade consequences [24]. Grade consequences were defined as a threat to course credit based on test performance. Those who listened to pre-selected “calm music” while studying had lower systolic blood pressure, lower heart rate, and higher test scores versus those who listened to “obnoxious music” [24]. However, the effects on heart rate and test scores were only significant for those whose course credit was threatened [24].

The objectives of this pilot study were to evaluate: (1) the impact of a classroom activity involving music (passively listening to music versus actively playing music) on anxiety associated with preparing for and taking an assessment; (2) the impact of the music activity on students’ future plans to use music as a stress-reducing mechanism; and (3) student level of enjoyment of the musical intervention. 

## 2. Materials and Methods

The Institutional Review Board (IRB) at the University of the Pacific approved this study. Participation in this project was voluntary and informed consent was obtained from all participants. This study took place within the Practicum 1 course, a required core course offered in the first semester of a three-year accelerated PharmD program. Practicum 1 is designed to develop pharmacy practice skills and knowledge through completion of self-study modules, attendance of lectures, and application of knowledge with guided practice simulations. The teaching methodology consists of a weekly one-hour lecture and a two-hour breakout session. Breakout sessions enable students to discuss pertinent information from the self-study modules and lectures, and provide students the opportunity to develop specific hands-on skills used in pharmacy practice. Weekly knowledge assessments are completed in each breakout session and count for the course participation grade.

This study took place within the smoking cessation module. The week before the study intervention, students received a one-hour lecture on smoking cessation counseling strategies. During the breakout session the following week, students were provided a table with all of the pharmacological smoking cessation aids. This was the students first time being exposed to this material in order to obtain consistency in baseline knowledge. This analysis focused on students “state-anxiety” as it relates to temporary anxiety provoked by a trigger; namely, the knowledge assessment on the smoking cessation aids. The following sections will describe in detail each of the assessment tools used, followed by an explanation of the procedures used and timeline for the study. 

### 2.1. Assessment Tools

An “Attitudes and Perceptions” survey was developed by investigators consisting of seven questions aimed to evaluate students’ previous experience with music, and attitudes and perceptions of using music for reducing test anxiety. Items 1–3 collected baseline characteristics, including individual’s information regarding typical amount of time spent listening to music per week, amount of time spent playing music per week and any prior musical training. The remaining four questions assessed students’ attitudes and perceptions regarding the use of music, both listening and playing, as a stress reducer before and after the music intervention. A 5-point Likert scale was used and coded numerically as follows: 1-strongly disagree, 2-disagree, 3-neutral, 4-agree, and 5-strongly agree. 

To assess student’s test anxiety throughout the study, The State-Trait Anxiety Inventory (STAI) tool was used. The STAI is commonly used in the clinical setting to distinguish anxiety from depressive symptoms. It has been used extensively and previously tested for both validity and reliability. The abbreviated short form tool was used in this study which contains 20-questions in total consisting of two subscales measuring (1) self-reported “trait-anxiety”, which measures general and long-standing anxiety (10 questions), and (2) “state anxiety” which is a temporary condition (10 questions) [25]. The response options for the “state-anxiety” scale assess the intensity of current feelings “at this moment” using the following scale: (1) not at all, (2) somewhat, (3) moderately so, and (4) very much so. Students completed all 20 questions but in evaluating anxiety as it relates to their current anxiety prior to an assessment, this study analyzed data from the “state-anxiety” subscale only. The range of possible scores for the “state-anxiety” subscale is 10 to 40 with higher scores indicating greater anxiety. Internal consistency estimates of reliability for the STAI scores (both state and trait) were obtained before conducting the main analyses. Based on Cronbach’s alpha (α = 0.90), a measure of internal consistency, for this sample of pharmacy students, the 10-item STAI state anxiety score was found to be reliable, strengthening the statistical conclusion validity of the study. 

As mentioned above, a knowledge assessment that counted toward student’s participation grade was used to serve as a tool to elicit test anxiety. This is a five-question multiple-choice knowledge assessment covering the material on the 2-page smoking cessation aid table provided to the students during the 25 min study period. Questions on the knowledge assessment administered at each of the 3 time-points were similar in difficulty level but different at each of the three time-points. The course coordinator created all of the questions and evenly divided them into each of the three time-point assessments based on difficulty level to assure consistency as best as possible. Note, however, the focus of this study was not on student performance on this knowledge assessment, but rather the test anxiety associated with taking this assessment.

### 2.2. Procedures

Students enrolled in the Practicum 1 course were randomly assigned to one of four lab sessions at the beginning of the semester. All four lab sessions occurred within the same week with a duration of 2 h each, taking place on both Tuesday and Thursday from 1–3 pm to 3–5 pm. On the given study day, each of the lab sessions were randomly divided into two groups in which one group was assigned to the active music playing group, and the other assigned to the passive listening music group. All students completed pre (Time-Point #1), mid (Time-Point #2) and post (Time-Point #3) measures. Time-point #1 served as the baseline, Time-Point # 2 served as the control and Time point #3 assessed the impact of the musical intervention. The overview of the measures and procedures can be found in Table 1 and are explained further below.

At the beginning of the lab session, Time-Point #1, students’ baseline was assessed, and all three assessment tools were completed including the attitudes and perceptions survey, STAI survey, and the smoking cessation knowledge assessment. Surveys were administered via Canvas, an online learning management system. Each measure administered was timed and released to students in sequential order to the entirety of the group. Students were asked to work independently and proctored by the course coordinator. Immediately following the allotted time to complete all three of the measures, students were given the study materials on the smoking cessation pharmacological aids to review for the following 25 min. Study materials consisted of the two-page table with all of the pharmacological smoking cessation aids approved by the Food and Drug Administration (FDA). This was the first time students were exposed to this material in order to measure a true baseline. 

Time-Point #2 served as the control to assess students’ state anxiety levels that accompany anticipated assessment without a musical intervention. After the 25 min of studying materials, students took the State-Trait Anxiety Inventory (STAI) survey, followed by a five-question smoking cessation knowledge assessment. 

After the knowledge assessment at time-point #2, students then broke off into their randomly assigned musical intervention group, either listening to music or actively playing music. Students were not allowed to choose whether or not they would prefer to actively play or passively listen. Those in the listening group were led to an outdoor space and instructed to listen to self-selected music via headphones for 25 min and overseen by teaching assistants. Students found a place in the grass to sit and individually listened to music for the allotted time. Students in the active music playing group were led to a separate outdoor area and played percussive instruments for 25 min. Percussive instruments were provided by the music therapy department. The students were led by music therapy graduate students and were asked to do a series of coordinated drumming exercises and at times played free-form. The two groups were entirely separated and not able to see or hear one another. 

At the end of the music interventions, Time-Point #3, served to measure the impact of the musical intervention. Both groups returned to the original classroom where they completed all three assessment tools in the order of survey on “Attitudes and Perceptions”, followed by STAI survey and lastly by the 5-question smoking cessation knowledge assessment. 

### 2.3. Statistical Analysis

A series of analyses (independent-samples t-tests, chi-squared tests of association, and Mann–Whitney tests for independent samples) were first conducted to verify that the random assignment to conditions had led to comparable groups at the start. A two-way ANOVA was next performed to address the first objective, treating the experimental condition (Listen/Play) as the between-subjects factor and time (Pre/Mid/Post) as the within-subjects factor. For the second objective, a two-way ANOVA was performed treating both time (Pre and Post) and type of usage (passive listening versus active playing) as within-subjects factors. In addition, simple main effects analyses were conducted when statistically significant interactions were detected. In addition to providing the frequency distribution based on the whole sample for responses related to the third study objective, an independent-samples t-test was employed to compare the experimental conditions. Post hoc power analyses and effect sizes were also estimated. Complete sets of data were available for all 202 study participants. The analyses were done with IBM SPSS Statistics version 26.0 (IBM Corp., Armonk, NY, USA). A *p*-value of less than or equal to 0.05 was considered significant.

## 3. Results

Findings on the comparability of students across the two conditions, the reliability of the measuring tool, and the results informing the research questions are presented below. Two hundred and two (202) students participated in the study, 99 students participated in the passive music arm, and 103 students in the active music arm. Despite random assignment, class scheduling issues resulted in there being unequal sizes across the conditions. 

Based on independent-samples t-tests, there was insufficient evidence to suggest the groups differed in their level of anxiety at the initial time-point. Based on chi-squared tests of association, there was insufficient evidence to suggest the distributions regarding gender or prior musical training differed between the groups. Mann–Whitney tests for independent samples were used to compare the groups in terms of the time spent playing or composing music and the time spent listening to music on a weekly basis, and the years of prior musical training. No statistically significant differences were found between all comparisons, (*p* > 0.05 for all tests). Thus, the selection threat to internal validity was not a concern.

The first objective was to evaluate the impact of a classroom activity involving music (passively listening to music versus actively playing music) on anxiety associated with preparing for and taking an assessment. To address the first study objective, the related two research questions are, “What effect does a classroom activity involving music have on anxiety associated with preparing for and taking an exam?” and “Does the change in anxiety across time differ between those who passively listen to music versus actively play music prior to an exam?” A two-way ANOVA was performed treating the experimental condition (Listen/Play) as the between-subjects factor and time (Pre/Mid/Post) as the within-subjects factor. See Table 2 for descriptive statistics. The main effect of condition was not statistically significant, F(1,200) < 1, *p* = 0.961, but the main effect of time was, F(1,200) = 141.935, *p* < 001. Further, the condition by time interaction approached statistical significance, F(1,200) = 3.463, *p* = 0.064. (Note: The conservative, lower-bound estimates were used because the sphericity assumption was not clearly met, *p* = 0.066). Simple main effects analyses suggest that, within the condition that just listened to music, state anxiety level, on average, was found to differ significantly between all three time points, initially rising from pre-test to mid-test (*p* = 0.017) and then decreasing at the post-test (*p* < 0.001) from the mid-test, as well as from the pre-test (*p* < 0.001). For the condition that played music, a similar rise and fall pattern was observed, but the initial rise was not significant (*p* = 0.433) although the decreased state anxiety from mid-test to post-test and from pre-test to post-test, were significant (*p* < 001, for both comparisons). If we ignore the marginally significant interaction and combine the two groups, focusing on the main effect of time, differences in state anxiety are found between all three time points (*p* = 0.025 comparing pre-test and mid-test means; *p* < 0.001 comparing pre-test and post-test means; and, and *p* < 0.001 comparing mid-test and post-test means). 

The second objective was to evaluate the impact of the music activity on students’ future plans to use music as a stress-reducing mechanism. To address the second study objective, the related two research questions are, “What effect does a classroom activity involving music have on students’ plans to use music as a stress-reducing mechanism?” and “Does the change across time in plans for using music to reduce stress depend on the way in which the student is thinking about incorporating it (passive listening versus active playing)?” A two-way ANOVA was performed treating both time (Pre and Post) and type of usage (passive listening versus active playing) as within-subjects factors. See Table 3 for descriptive statistics. Means are based on combining responses that students gave for two statements (1 and 2, pre-mean; 3 and 4, post-mean). The main effect of time, F(1,201) =288.518, *p* < 0.001, type of usage, F(1,201)= 203.361, *p* < 0.001, and the type by time interaction F(1,201) = 19.207, *p* < 0.001 were all statistically significant. The average level of agreement for statements about using music to reduce stress increased over time; moreover, the agreement increased more so in terms of planning to actively play music compared to passively listen to music. The simple main effects analyses confirmed that the increases over time were statistically significant for both types of usage; also, at both time points, there was a stronger agreement for statements about listening to music than for those about playing music.

The third study objective was to evaluate student level of enjoyment of the musical intervention. Considering all students together, regardless of music condition assigned, only 7% disagreed or strongly disagreed, 12% were neutral, and over 80% agreed or strongly agreed with the statement, “This music experience prior to taking an exam was enjoyable.” The average level of agreement was significantly higher for the passive listening (M = 4.23, SD = 0.75) than the active playing (M = 3.85, SD = 1.00) condition, t(189.027) = 3.034, *p* = 0.003.

A post hoc power analysis was conducted, as show in Table 4.

## 4. Discussion

The goal of healthcare education is to provide students with the knowledge and abilities to provide healthcare and pass licensure; however, it is evident that such rigorous training can come at a cost. Especially for those in an accelerated program as Frick et al. concluded that students in a three-year PharmD program with a unique educational model experienced more stress compared to students in a traditional four-year PharmD program [26]. Stress, anxiety, and depression prevent professional students from performing at their highest potential academically and subsequently, the quality of patient care may be reduced due to distressed clinicians [15,27]. As research confirms the link of stress and its negative effect on academic performance, professional schools should put more efforts into helping students become more self-aware of their stress and develop coping strategies [27]. Previous studies have shown that psychological intervention helped significantly reduce scores of test anxiety, psychological distress, and lack of motivation, while helping to improve students’ GPA within a pharmacy program [28]. There is a call for institutional changes to help reduce stress and provide students with appropriate coping mechanisms [29]. 

This study is the first of its kind exploring the effect of music interventions on test anxiety among pharmacy students. This study showed that regardless of whether students listened to music or played music, music reduced test anxiety. A simple strategy such as listening to music prior to a triggering event such as a knowledge test could be a useful coping strategy for students who experience situational anxiety. In addition, the high levels of agreement with the statement that asked about the plan to use music for reducing stress in the future suggest that the experimental conditions may have increased students’ perceptions that music can be useful for reducing test anxiety. 

In preparing students to take on the roles and responsibilities of healthcare providers, it is important for educators to provide an environment for reduced stress during their didactic training as well as equip students with stress-reducing strategies to be used as future professionals. While the use of music did reduce test anxiety in this study, there were several limitations to note. The knowledge assessment that was administered is used for the weekly participation grade. Although submission is mandatory to pass the course, full credit is given regardless of the score. It is possible the low stakes nature of the knowledge assessment did not elicit the same amount of anxiety that a higher stakes graded assessment typically would. This may have resulted in a decreased level of anxiety as the study progressed. The study was designed to have time-point #2 serve as the control where students completed the assessment methods without the use of the musical intervention. Doing so allowed for a greater sample size in each of the two music intervention arms. However, students having to repeat the knowledge assessment three times may have contributed to the decrease in anxiety throughout the study. Future studies should consider adding a third group as the control group instead of repeated testing in order to explore this potential limitation. 

Previous research has shown that the most important factor in reducing stress was the degree of liking the music, supporting the decision for the music to be self-selected within the passive listening music intervention. [30,31,32] However, students were randomized into two different music conditions and not able to choose whether or not they would prefer to actively play or passively listen. Perhaps self-selection into the music intervention group itself would have resulted in a greater impact on stress reduction as well. This was a one-time music intervention that lasted a very short period of time. Given the brief intervention, results showed that it did have a positive effect on reducing anxiety, but it would be beneficial to see the effect of repeated musical interventions over a longer period to rule out the novelty effect threat to external validity. In addition, this study only used subjective measures, including the attitudes and perceptions survey and the STAI survey. 

There are some limitations that may have contributed to the finding that the average level of agreement was higher for the passive listening than the active playing condition when students responded to the item, “This music experience prior to taking an exam was enjoyable.” Each active music condition group consisted of 25 students in a drum circle, which may have been overwhelming to students who are uncomfortable playing music, especially in front of others. Additionally, despite music therapy students leading the group, at times, the directions given caused auditory chaos with students doing a freeform playing. Perhaps more direction and guidance would provide students with increased confidence to play their instrument. Providing students with a specific rhythm to follow at particular times may have increased their confidence, ultimately reducing stress. Furthermore, if students felt inadequate at playing their instrument, this may have increased their anxiety level. 

Additionally, this study did not focus on the effects of music intervention on student performance. Future research should use a validated knowledge assessment tool to determine if the use of music can improve academic performance. It would be interesting to determine whether the impact of music on academic performance varies by level of cognitive demand (e.g., knowledge, comprehension, and evaluation). The use of objective measures such as heart rate, blood pressure, and cortisol levels could also be explored. Future research should use higher stakes assessments and measure the long-term effects of musical interventions on both state anxiety as well as the academic performance with the use of more longitudinal musical interventions. Assessing the impact of using music throughout the curriculum with repeated exposure should also be completed.

## 5. Conclusions

Rigorous and challenging health care programs expose students to high levels of anxiety that can undermine their ability to learn and perform well on tests. Incorporating effective ways to deal with stress into health care curriculums is imperative for student success and future career satisfaction. This study examined the use of music as an innovative coping method to address test anxiety. The use of music appears to have helped reduce test anxiety, even after one brief musical intervention, regardless of whether students passively listened to music or actively played music. This inquiry into the use of music as an instrument to help decrease stress and anxiety demonstrates that this could be a promising area of future research and an important addition to a health care professional school curriculum.

## Figures and Tables

**Table 1:** Timeline of Study Procedures and Assessment Tools Used.

Header	Pre (Time Point #1)	Mid (Time Point #2)		Post (Time Point #3)	
Duration (Minutes)	20	25	15	25	20
Procedures/Measures	Participants Completed: -Perceptions and Attitudes Survey -STAI Survey -Knowledge Assessment	Participants provided study material to independently review smoking cessation aids	Participants Completed: -STAI Survey -Knowledge Assessment	Study Intervention: Participants either passively listened to self-selected music OR Actively played music in drum circle	Participants Completed: -Perceptions and Attitudes Survey -STAI Survey -Knowledge Assessment

STAI = State Trait Anxiety Inventory.

**Table 2:** Descriptive Statistics for State Anxiety over Time and by Musical Intervention.

	Both Conditions (*n* = 202)		Passive Listening to Music (*n* = 99)		Active Playing of Music (*n* = 103)	
Time Point	*M*	*SD*	*M*	*SD*	*M*	*SD*
Pre (Time Point #1)	22.28	6.31	22.35	6.33	22.20	6.33
Mid (Time Point #2)	23.15	6.68	23.69	6.92	22.63	6.44
Post (Time Point #3)	16.78	5.39	16.22	4.91	17.32	5.80

M = Mean; SD = Standard Deviation.

**Table 3:** Descriptive statistics regarding the agreement with statements about using music to reduce stress over time and by type of music usage ^a^.

	Both Conditions (*n* = 202)		Passive Listening to Music (*n* = 99)		Active Playing of Music (*n* = 103)	
Time Point	*M*	*SD*	*M*	*SD*	*M*	*SD*
Pre (Time Point #1)	2.70	0.93	3.31	1.20	2.08	1.07
Post (Time Point #3)	3.60	0.85	4.01	0.91	3.19	1.13

^a^ Statements: 1. I use music (listening) as an anxiety reducer prior to exams (Pre mean); 2. I use music (playing, composing) as an anxiety reducer prior to exams (Pre mean); 3. I would use music (listening) as anxiety reducer prior to exams in the future (Post mean); 4. I would use music (playing, composing) as anxiety reducer prior to exams in the future (Post mean). M = Mean; SD = Standard Deviation.

**Table 4:** Observed power and partial eta squared effect sizes for the main analyses.

Study Objective	Analysis	Effect	Observed Power	Partial Eta Squared
#1	Two-way ANOVA (between and within-subjects factors)	A: Condition	0.050	0.000
		B: Time	1.000	0.415
		A × B Interaction	0.457	0.017
#2	Two-way ANOVA (within-subjects factors)	A: Type of Usage	1.000	0.503
		B: Time	1.000	0.589
		A × B Interaction	0.992	0.087
#3	Independent- samples t-test	A: Condition	0.851	0.044

## Data Availability

The data presented in this study are available on request from the corresponding author.
